# Multifunctional MXene for Thermal Management in Perovskite Solar Cells

**DOI:** 10.1007/s40820-025-01855-5

**Published:** 2025-08-04

**Authors:** Zhongquan Wan, Runmin Wei, Yuanxi Wang, Huaibiao Zeng, Haomiao Yin, Muhammad Azam, Junsheng Luo, Chunyang Jia

**Affiliations:** 1 National Key Laboratory of Electronic Films and Integrated Devices, School of Integrated Circuit Science and Engineering, University of Electronic Science and Technology of China, Chengdu, 611731 People’s Republic of China; 2 Shenzhen Institute for Advanced Study, University of Electronic Science and Technology of China, Shenzhen, 518110 People’s Republic of China

**Keywords:** Perovskite solar cells, Heat accumulation, Thermal management, Multifunctional MXene, Defect passivation

## Abstract

Perovskite solar cells (PSCs) have emerged as promising photovoltaic technologies owing to their remarkable power conversion efficiency (PCE). However, heat accumulation under continuous illumination remains a critical bottleneck, severely affecting device stability and long-term operational performance. Herein, we present a multifunctional strategy by incorporating highly thermally conductive Ti_3_C_2_T_X_ MXene nanosheets into the perovskite layer to simultaneously enhance thermal management and optoelectronic properties. The Ti_3_C_2_T_X_ nanosheets, embedded at perovskite grain boundaries, construct efficient thermal conduction pathways, significantly improving the thermal conductivity and diffusivity of the film. This leads to a notable reduction in the device’s steady-state operating temperature from 42.96 to 39.97 °C under 100 mW cm^−2^ illumination, thereby alleviating heat-induced performance degradation. Beyond thermal regulation, Ti_3_C_2_T_X_, with high conductivity and negatively charged surface terminations, also serves as an effective defect passivation agent, reducing trap-assisted recombination, while simultaneously facilitating charge extraction and transport by optimizing interfacial energy alignment. As a result, the Ti_3_C_2_T_X_-modified PSC achieve a champion PCE of 25.13% and exhibit outstanding thermal stability, retaining 80% of the initial PCE after 500 h of thermal aging at 85 °C and 30 ± 5% relative humidity. (In contrast, control PSC retain only 58% after 200 h.) Moreover, under continuous maximum power point tracking in N_2_ atmosphere, Ti_3_C_2_T_X_-modified PSC retained 70% of the initial PCE after 500 h, whereas the control PSC drop sharply to 20%. These findings highlight the synergistic role of Ti_3_C_2_T_X_ in thermal management and optoelectronic performance, paving the way for the development of high-efficiency and heat-resistant perovskite photovoltaics.

**Supplementary Information:**

The online version contains supplementary material available at 10.1007/s40820-025-01855-5.

## Highlights

- Incorporating Ti_3_C_2_T_X_ nanosheets enhanced perovskite thermal conductivity (from 0.236 to 0.413 W m^−1^ K^−1^) and reduced operating temperature by ~3 °C under illumination, mitigating heat-induced degradation.
- Ti_3_C_2_T_X_ offers multiple additional functionalities, including defect passivation, improved charge transfer efficiency, and optimized energy level alignment.
- Champion power conversion efficiency (PCE) reached 25.13% (vs. 23.70% control). Retained 80% PCE after 500 h at 85 °C/RH = 30 ± 5%, outperforming control (58% after 200 h). MPP tracking showed 70% PCE retention after 500 h in N_2_ (vs. 20% control).

**Supplementary Information:**

The online version contains supplementary material available at 10.1007/s40820-025-01855-5.

## Graphical abstract

**Figure:**
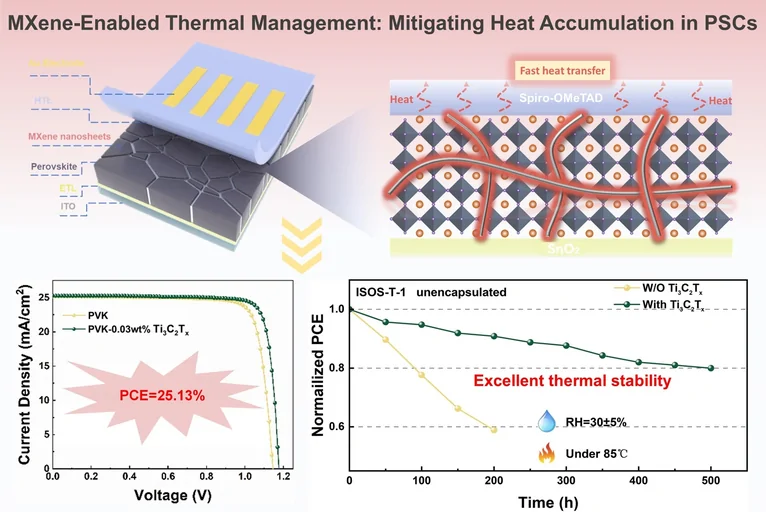

## Introduction

Photovoltaic technology, which directly converts solar energy into electrical energy, is considered one of the most promising and economically viable renewable technologies. Organic–inorganic hybrid perovskite solar cells (PSCs) have achieved rapid development over the past decade and have garnered significant attention due to excellent optoelectronic properties [1–6]. These properties include a broad spectral response range, high light absorption coefficient, low exciton binding energy, long carrier diffusion length, and high carrier mobility. To date, the highest certified power conversion efficiency (PCE) of PSCs has reached 26.7%, which is almost comparable to most advanced levels of crystalline silicon solar cells [7–10]. However, the limited lifespan of PSCs under harsh conditions such as high humidity, elevated temperatures, and prolonged illumination has become a major obstacle to their commercialization [6, 11, 12].

Although encapsulation technologies can effectively protect against moisture and oxygen [13–16], the issue of thermal stability remains a core challenge that urgently needs to be addressed. In practical applications, the operating temperature of PSCs often increases due to sunlight exposure and rising ambient temperatures, especially under concentrated light or high-temperature conditions where the temperature can exceed 85 °C. This can lead to local strain, lattice mismatch, and degradation of perovskite, significantly impairing the PCE of PSCs [17–19]. Even when the temperature returns to room temperature, the photovoltaic performance of PSCs is difficult to recover. Meanwhile, with the temperature gradually rising, there is always a reversible PCE reduction, which is one universal phenomenon regardless of compositions and configurations for PSCs, owing to the increased Urbach energy and scattering interaction of electrons with phonons [20–22]. Therefore, accelerating heat dissipation to reduce the operating temperature of PSCs is crucial for maintaining the maximum power output and long-term stability [23]. As for heat dissipation, efficient thermal conduction and radiation are needed to accelerate heat transfer from within PSCs to the external environment, which is closely linked to the thermal properties of each functional layer within PSCs [24].

Research has shown that over 90% of waste heat in PSCs is generated by the perovskite layer due to its photoelectric conversion characteristics [23]. However, the perovskite material itself has an extremely low thermal conductivity (around 0.2 W m^−1^ K^−1^), making it difficult for heat to dissipate effectively within the PSC. This leads to continuous temperature buildup, which further deteriorates the performance of PSCs. Thermal management technologies are currently widely used to regulate heat flow in electronic devices, enhancing their operational stability and reliability [25, 26]. These techniques have been widely applied in silicon-based solar cells, perovskite lasers, and perovskite light-emitting diodes [27, 28]. However, despite success in these areas, the application of thermal management technologies in PSCs is still relatively limited. To date, only a few studies have attempted to incorporate highly thermally conductive materials into PSCs to improve thermal management performance. For example, the introduction of highly conductive 2D hexagonal boron nitride nanosheets [29] into the perovskite layer or the addition of materials such as aluminum oxide [30], silicon dioxide [31], and zeolite [32] into hole transport layer has shown significant potential for improving heat transfer capabilities [33–36]. However, due to insulating properties, these materials have not significantly improved charge transport or reduced defects within the PSCs, thus limiting the overall performance enhancement of PSCs.

Compared to other materials, MXene as a new type of 2D material has attracted widespread attention due to the excellent metallic conductivity, high chemical stability, and thermal conductivity [37, 38]. Specifically, Ti_3_C_2_T_X_ MXene has a multilayer structure that exhibits an impressive thermal conductivity of up to 55.8 W m⁻^1^ K⁻^1^ (Fig. S1) [39], surpassing existing thermal management materials like h-BN, Al_2_O_3_, SiO_2_, and zeolite (Table S1). This makes it one of the most effective thermally conductive materials. Beyond its thermal properties, Ti_3_C_2_T_X_ also plays multiple critical roles in PSCs, such as optimizing work function (*W*_F_) for better energy level alignment, and defect passivation [40–46]. These multifunctional advantages highlight the great potential of Ti_3_C_2_T_X_ for constructing PSCs with not only high power conversion efficiency but also excellent thermal stability. However, its specific role in improving the thermal management of PSCs remains underexplored and merits systematic investigation.

**Fig. 1:**
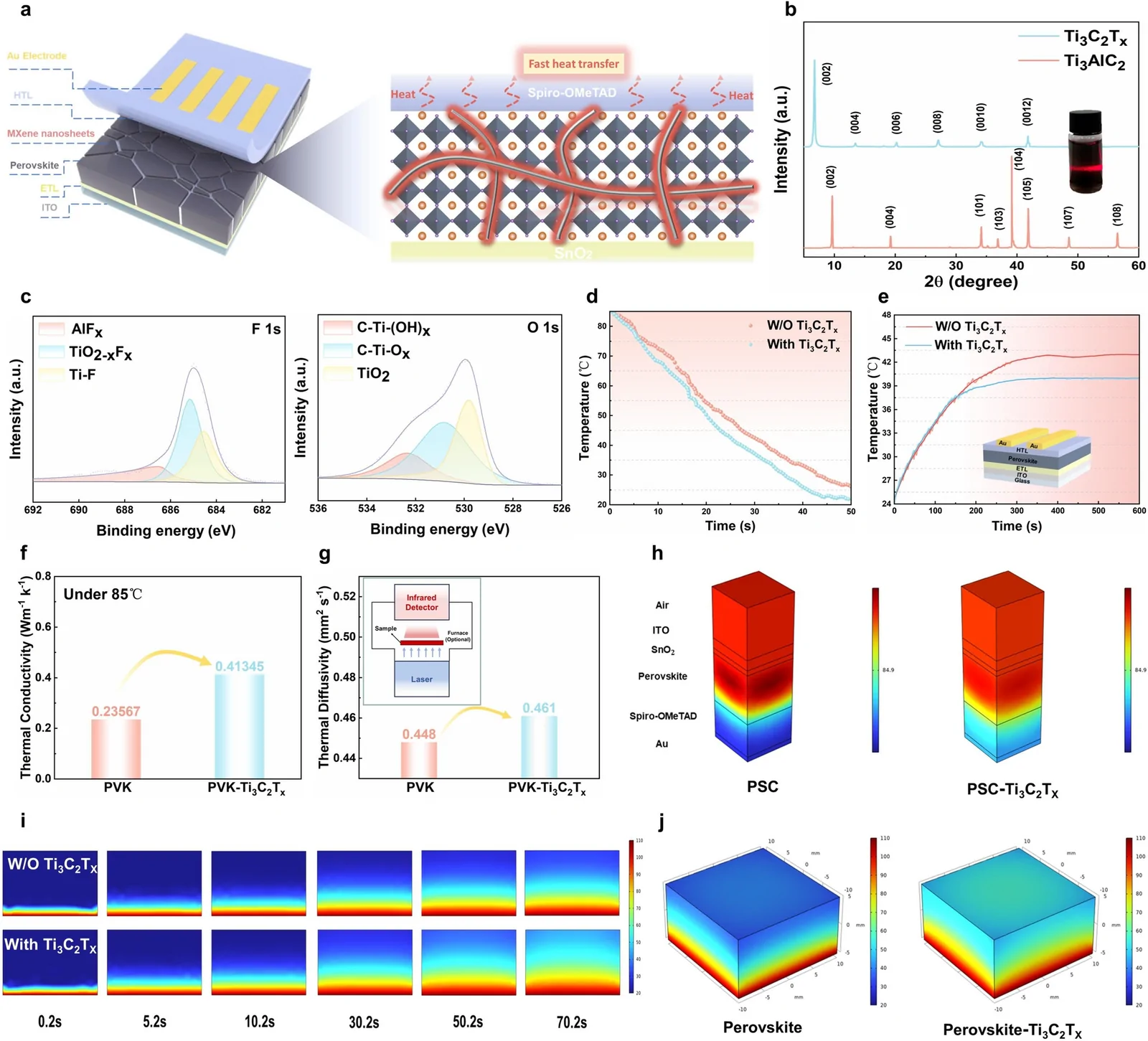
**a** Schematic diagram of efficient heat transfer pathways formed by Ti_3_C_2_T_X_ nanosheets within the perovskite layer. **b** XRD patterns of Ti_3_AlC_2_ and the Ti_3_C_2_T_X_ with the inset illustrating the image of Ti_3_C_2_T_X_-DMF dispersion. **c** High-resolution XPS spectra of F 1*s* and O 1*s* for Ti_3_C_2_T_X_. **d** Real-time temperature tracking of perovskite films during the cooling test. **e** Time-dependent temperature evolution of control and Ti_3_C_2_T_X_-modified PSCs under AM 1.5G standard illumination at 100 mW cm^−2^. **f** Thermal conductivity of perovskite with and without Ti_3_C_2_T_X_ under 85 °C. **g** Thermal diffusion coefficients of perovskite films with and without Ti_3_C_2_T_X_. **h** Internal temperature distribution of PSCs with and without Ti_3_C_2_T_X_ modification. **i** Transient temperature distribution during the annealing process of perovskite films. **j** Steady-state temperature distribution during the annealing process of perovskite films

Herein, we introduce highly thermally conductive Ti_3_C_2_T_X_ nanosheets into the perovskite layer for the first time, serving as multifunctional additives to simultaneously enhance heat dissipation and optoelectronic properties. By forming efficient thermal conduction pathways at perovskite grain boundaries, Ti_3_C_2_T_X_ effectively suppresses heat accumulation within PSCs, reducing the steady-state operating temperature from 42.96 to 39.97 °C under AM 1.5G illumination at 100 mW cm^−2^ (Fig. 1a). Beyond thermal management, Ti_3_C_2_T_X_ also chemically interacts with undercoordinated Pb^2+^, enabling defect passivation within the perovskite film. Additionally, its tunable *W*_F_ improves energy level alignment at the perovskite/transport layer interfaces, thereby facilitating more efficient charge extraction and transfer. These synergistic effects contribute to a significantly enhanced device performance, with the Ti_3_C_2_T_X_-modified PSC delivering a champion PCE of 25.13%. Remarkably, the Ti_3_C_2_T_X_-modified PSC maintain 80% of its initial efficiency after 500 h of thermal aging under ISOS-T-1 conditions (85 °C, RH = 30 ± 5%) without encapsulation. Furthermore, under continuous maximum power point (MPP) tracking in N_2_ atmosphere, the PSC exhibits a 3.5-times improvement in operational stability compared to the control PSC, retaining 70% of its initial PCE after 500 h. These results demonstrate that incorporating Ti_3_C_2_T_X_ offers a promising strategy toward the development of high-efficiency and heat-resistant perovskite photovoltaics.

## Experimental Section

### Materials

Formamidinium iodide (FAI, 99.99%), MABr (99.99%), PbI_2_ (99.99%), PbBr_2_ (99.99%), Spiro-OMeTAD, and 4-tert-butylpyridine (tBP) were purchased from Xi'an Polymer Light Technology Corp. The N,N-dimethylformamide (DMF), dimethyl sulfoxide (DMSO), bis(trifluoromethane)sulfonimide lithium salt (Li-TFSI), and chlorobenzene (CB) were purchased from Sigma-Aldrich. All chemicals and solvents were obtained from commercial sources with certified purity and used as received without further purification.

400 mesh Ti_3_AlC_2_ powder was purchased from Jilin 11 Technology Co., Ltd. Hydrochloric acid (37 wt%), hydrofluoric acid (40 wt%), lithium chloride, ethylenediamine, N,N′-methylenebis(acrylamide), and poly(ethylene glycol) diacrylate were purchased from Shanghai Aladdin.

### Preparation of Ti_3_C_2_T_X_ Dispersion in DMF

Ti_3_C_2_T_X_ MXene was prepared using an improved method. Specifically, 20 mL of HF (40 wt%) and 20 mL of HCl (37 wt%) were poured into a polytetrafluoroethylene (PTFE) beaker and thoroughly stirred. Then, 2 g of Ti_3_AlC_2_ powder was added to the PTFE beaker and stirred while heating at 45 °C for 12 h. After etching, the mixture was centrifuged and washed several times with distilled water (3500 rpm, 1 min) until the pH of the supernatant approached neutral (pH > 6). The collected precipitate was then added to a beaker containing 40 mL of distilled water and 2 g of LiCl, and stirred at 45 °C for 12 h. Subsequently, Ti_3_C_2_T_X_ MXene was delaminated through several rounds of centrifugation (5000 r min^−1^, 1 min). The precipitate was transferred to 40 mL of distilled water and then sonicated for 2 h under argon bubbling. After sonicating, the mixture was centrifuged at 8000 r min^−1^ for 1 h, and the precipitate was washed with anhydrous DMF several times to remove residual water. The resulting Ti_3_C_2_T_X_ MXene was diluted to 0.5 mg mL^−1^ for subsequent experiments.

### Device Fabrication

Perovskite precursor was prepared by dissolution 1097.20 mg of PbI_2_, 154.14 mg of PbBr_2_, 380.24 mg of FAI, and 43.68 mg of MABr in 2 mL DMF and DMSO (the volume ratio of 4:1) mixing solution. 103.92 mg of CsI was dissolved in 200 μL DMSO. After fully stirring of the two solution, 68 μL CsI precursor was added to the perovskite precursor solution and then continue stirring. Add different weight percent of the Ti_3_C_2_T_X_-DMF dispersion to the prepared precursor solution to create a perovskite precursor solution containing Ti_3_C_2_T_X_ and stir it for two hours.

Spiro-OMeTAD solution: dissolved 72.3 mg of Spiro-OMeTAD in 1 mL of chlorobenzene, then add 28.8 µL of tBP and 17.5 µL of Li-TFSI solution (104 mg of Li-TFSI in 200 µL of acetonitrile), and stirred constantly.

Glass/ITO substrates were cleaned with isopropyl alcohol, ethyl alcohol, and deionized water in an ultrasonic bath for 30 min, respectively, and then dried by flowing nitrogen gas. A 1 mL electron transport layer (ETL) solution was prepared by mixing SnO_2_ colloidal dispersion with deionized water at a volume ratio of 1:3. The SnO_2_ film was then deposited by spin-coating the solution at 3000 r min^−1^ for 30 s with an acceleration of 500 (r min^−1^) s^−1^. The ITO/SnO_2_ substrates were then treated with ultraviolet/ozone for 15 min and promptly transferred into a nitrogen-filled glovebox for subsequent processing. The perovskite film was deposited on the treated ITO/SnO_2_ substrates by spin-coating filtered perovskite precursor at the speed of 1300 r min^−1^ for 10 s and 5000 r min^−1^ for 45 s with an acceleration of 500 (r min^−1^) s^−1^. During the process of spin-coating, 200 μL CB was dropped at the center of the substrates at the last 15 s, and then followed by 110 °C heating for 60 min. The HTL was spin-coated on the surface of perovskite films with the speed of 3000 r min^−1^ for 30 s with an acceleration of 1000 (r min^−1^) s^−1^. In the end, the Au electrode was deposited by vacuum thermal evaporation, and the active area is 0.07 cm^2^ for each PSC.

### Characterizations

The morphology of Ti_3_C_2_T_X_ nanosheets was observed by transmission electron microscopy (TEM) (JEOL, JEM-F20, Japan). X-ray photoelectron spectroscopy (XPS) (Thermo Scientific, K-Alpha, USA) and X-ray diffractometry (XRD) (Rigaku, MiniFlex 600, Japan) were used for the determination of elemental valence and the crystal structure of the Ti_3_C_2_T_X_ nanosheets.

The surface temperature tracking was determined by an infrared camera (Jugo Electronics, China).Thermal conductivity of perovskite was determined using a thermal conductivity analyzer (Hot Disk, TPS 2500S, Sweden). Thermal diffusion coefficients of perovskite films were determined using a laser thermal conductivity meter (NETZSCH LFA 457 MicroFlash/NETZSCH LFA 467).The surface, cross-sectional morphologies, and elemental distribution of perovskite films were observed by scanning electron microscope (SEM) analysis (JEOL JSM-7600F) equipped with EDS (Thermo Scientific, Apreo 2S, USA). The X-ray diffraction (XRD) measurement of perovskite film was performed by Panalytical X’ Pert PRO with Cu Kα radiation. Water-contact angles of Ti_3_C_2_T_X_-modified perovskite films were examined by drop shape analyzer (Krüss DSA100). The photovoltaic and EIS analysis were performed by using electrochemical workstation (CHI 760E, Shanghai Chenhua) and solar simulator (Sirius-SS150A-D, Zolix Instruments Co. Ltd., Beijing, China) in ambient air condition. The voltage sweep range of current–voltage characteristics is 0 ~ 1.2 V, the sweep speed is 0.2 V s^−1^, and the dwell time is 2 s. Before analysis, the light source was precisely calibrated by standard Si solar cell. The EIS data were fitted by ZView equivalent circuit. The external quantum efficiency (EQE) spectra were recorded by QTest Station 2000 IPCE Measurement System (CROWNTECH, USA). The ultraviolet photoemission spectroscopy (UPS) measurements were performed by Thermo Fisher ESCALAB 250Xi. The atomic force microscope (AFM) measurements were performed by Bruker Dimension Icon. The steady-state photoluminescence (PL) spectra were measured by HITACHI (model F-4600) spectrophotometer with the excitation wavelength of 460 nm. The time-resolved PL (TRPL) spectra were measured at room temperature using of time-correlated single photon counting (TCSPC) technique with an excitation wavelength of 474 nm and an emission wavelength of 785 nm. The instrument for TRPL measurement was FluoroLog-3 Modular spectrofluorometer (HORIBA Jobin Yvon).

## Results and Discussion

### Synthesis and Characterization of Ti_3_C_2_T_X_ Nanosheets

Ti_3_AlC_2_ is a typical MAX phase material, with Ti (M element) layers alternating with Al (A element) layers, and C (X element) atoms filling the octahedral sites between the Ti layers [45]. Using Ti_3_AlC_2_ as the starting material, we successfully prepared Ti_3_C_2_T_X_ nanosheets dispersed in DMF through etching, exfoliation, and ultrasonic treatment. To confirm the successful conversion of Ti_3_AlC_2_ to Ti_3_C_2_T_X_, XRD was performed on both the original Ti_3_AlC_2_ and the Ti_3_C_2_T_X_ (Fig. 1b). The XRD patterns showed that the strongest diffraction peak (104) of Ti_3_AlC_2_ disappeared, and the diffraction peaks of MAX phase (002) (2θ ≈ 9.5°) and (004) (2θ ≈ 19.2°) shifted toward lower angles. The diffraction peaks corresponding to (002), (004), (006), and (008) crystal planes of Ti_3_C_2_T_X_ appeared at 7°, 14°, 22°, and 28°, respectively, consistent with previous studies [13]. These results confirm the complete conversion of Ti_3_AlC_2_ into Ti_3_C_2_T_X_. Additionally, the dispersion of Ti_3_C_2_T_X_ in DMF shown in the inset of Fig. 1b exhibited evident Tyndall effect, demonstrating the good dispersibility of Ti_3_C_2_T_X_ nanosheets in DMF.

For more intuitive structural information, we observed the Ti_3_C_2_T_X_ nanosheets using TEM, which revealed that the lateral size of the Ti_3_C_2_T_X_ nanosheets is approximately 800 nm (Fig. S2). We then analyzed the surface elemental composition of Ti_3_C_2_T_X_ using X-ray photoelectron spectroscopy (XPS). XPS spectra detected Ti, C, O, and F elements, while no Al was detected (Fig. S3), indicating that the metal bonds connecting the MAX phase had been completely etched. The high-resolution XPS spectra of each element are shown in Fig. S4. It was found that the Ti 2*p* peaks at the binding energies of 455.08 (460.88), 455.50 (461.58), 456.60 (462.78), and 458.88 (464.60) eV corresponded to Ti–C, Ti–O, Ti–OH, and TiO_2_, respectively. The C 1*s* peak at 282.00 eV attributed to Ti–C in Ti_3_C_2_T_X_, with two additional peaks corresponding to C–C and C–O. In Fig. 1c, the F 1*s* peaks at 684.60 and 685.20 eV corresponded to Ti–F and TiO_2-X_F_X_, while the peak at 686.60 eV was associated with trace etching byproducts, AlF_X_. In O 1*s* spectra, the peaks at 529.80, 531.00, and 532.88 eV were attributed to TiO_2_, C–Ti–O_X_, and C–Ti–(OH)_X_, respectively. Based on the above analysis, we confirmed that the primary termination groups of prepared Ti_3_C_2_T_X_ are O, OH, and F. The termination groups of Ti_3_C_2_T_X_ play a critical role in its properties. Furthermore, XPS analysis reveals that the prepared Ti_3_C_2_T_X_ exhibits moderate oxidation, which is consistent with previous studies and contributes to its excellent properties. This partial oxidation leads to the formation of Ti–O bonds or TiO_2_ nanoparticles, gradually shifting the material from metallic to semiconducting behavior. Such a transition can be beneficial for photovoltaic performance by tuning the work function and improving energy level alignment. However, excessive oxidation may impair the intrinsic electrical and thermal conductivity of Ti_3_C_2_T_X_, compromising its thermal management function. Therefore, a controlled degree of oxidation may play a key role in balancing thermal and electronic properties, contributing to the overall efficiency and stability of PSCs [47, 48].

### Improving Heat Transfer within the Perovskite Layer

Previous studies have shown that waste heat in perovskite layer mainly originates from the energy released when carriers are trapped by defects [23]. Furthermore, due to the ionic nature of hybrid perovskite, most defects in polycrystalline film are concentrated at grain boundaries, leading to a higher local temperature at boundaries compared to other regions. Simultaneously, phonon scattering and defect scattering are more severe at grain boundaries, which impede heat conduction and cause heat accumulation, leading to a continuous temperature rise in perovskite layer [49]. To address this issue, we introduced multifunctional Ti_3_C_2_T_X_ nanosheets with high thermal conductivity into perovskite layer. The metal carbides within Ti_3_C_2_T_X_ provide excellent thermal conductivity through strong covalent bonding. The schematic diagram illustrates that Ti_3_C_2_T_X_ nanosheets are uniformly distributed at grain boundaries of perovskite layer, forming efficient heat conduction pathways that accelerate heat transfer, mitigating heat accumulation (Fig. 1a). Next, we conducted experiments to assess the enhancement of heat transfer in perovskite layer due to the addition of Ti_3_C_2_T_X_. In all perovskite samples containing Ti_3_C_2_T_X_, the weight percent of Ti_3_C_2_T_X_ was consistently set at 0.03 wt%, which was identified as the optimal concentration in subsequent experiments.

To reveal the impact of Ti_3_C_2_T_X_ on thermal conductivity of perovskite films, we used an infrared thermal imager to monitor the natural cooling process of perovskite films from 85 to 25 °C under room temperature with 30 ± 5% relative humidity. We prepared perovskite films with and without Ti_3_C_2_T_X_, heating them on a hot plate until their surface temperature reached 85 °C, and then quickly transferred to a room-temperature cooling platform (Fig. S5). Within the same cooling period, the Ti_3_C_2_T_X_-modified film exhibited lower temperatures compared to control film, indicating a higher heat exchange efficiency with environment (Fig. S6). To further quantify the cooling rate, real-time temperature tracking was performed using the same infrared imaging technique during cooling process. As shown in Fig. 1d, the temperature decay rate of Ti_3_C_2_T_X_-modified film was significantly faster, consistent with the typical Fourier law of heat conduction, aligning with the observed cooling phenomena.

Additionally, we monitored the surface temperature of PSC in real time under AM 1.5G standard illumination (100 mW cm^−2^) at room temperature and a relative humidity of 30 ± 5% (Fig. 1e). The results showed that Ti_3_C_2_T_X_-modified PSC had a reduced stable operating temperature by approximately 3 °C compared to control PSC (from 42.96 to 39.97 °C), due to faster internal heat transfer to surrounding areas. Given the negative correlation between power output and temperature in PSCs, the reduction of operating temperature is undoubtedly beneficial for maintaining high efficiency. To further elucidate the intrinsic mechanism of Ti_3_C_2_T_X_ for heat dissipation improvement of perovskite, we used the Hot Disk method to test the thermal conductivity of perovskite with and without Ti_3_C_2_T_X_ at 85 °C. The results showed that the introduction of Ti_3_C_2_T_X_ increased the thermal conductivity of perovskite from 0.236 to 0.413 W m⁻^1^ K⁻^1^ (Fig. 1f), indicating a significant improvement in heat transfer performance. Similarly, we measured the thermal diffusivity of perovskite films with and without Ti_3_C_2_T_X_ using a laser flash apparatus. The thermal diffusivity of films was calculated by applying a laser pulse to heat the bottom of film and monitoring the temperature change at the top of film with an infrared detector. The results showed that the thermal diffusivity of perovskite film increased by 0.013 after modifying with Ti_3_C_2_T_X_ (Fig. 1 g). These findings provide strong evidence that Ti_3_C_2_T_X_ serves as an effective heat transfer pathway in perovskite film, significantly enhancing heat dissipation efficiency and contributing to the long-term stability of PSCs.

To gain deeper insight into the heat transfer mechanisms within perovskite film, we employed finite element analysis (FEA) to simulate the heat transfer process in PSCs [50]. We coupled the semiconductor and heat transfer in solids modules to simulate the temperature distribution within PSCs operating under standard AM 1.5G illumination in a confined indoor environment without airflow, with an initial ambient temperature set to 40 °C. Additional key input parameters are listed in Table S2. Ti_3_C_2_T_X_-modified PSCs exhibited faster heat conduction rates, and the temperature of perovskite layer was significantly reduced, which provides reliable support for the long-term stability of PSCs (Fig. 1 h).

In addition, we simulated the annealing process of perovskite films. As depicted in Fig. 1i, j, after introducing Ti_3_C_2_T_X_, the longitudinal temperature gradient during the annealing process was significantly reduced. Previous studies have shown that delayed surface heating and uneven internal temperature distribution can lead to a decrease in crystallization quality, but the introduction of Ti_3_C_2_T_X_ effectively mitigated this issue. The simulation results, along with the experimental findings, jointly confirmed the advantage of Ti_3_C_2_T_X_ in improving heat transfer in perovskite layer, further demonstrating its potential in enhancing the thermal stability of PSCs.

### Multifunctional Effect of Ti_3_C_2_T_X_ on Perovskite Layer

**Fig. 2:**
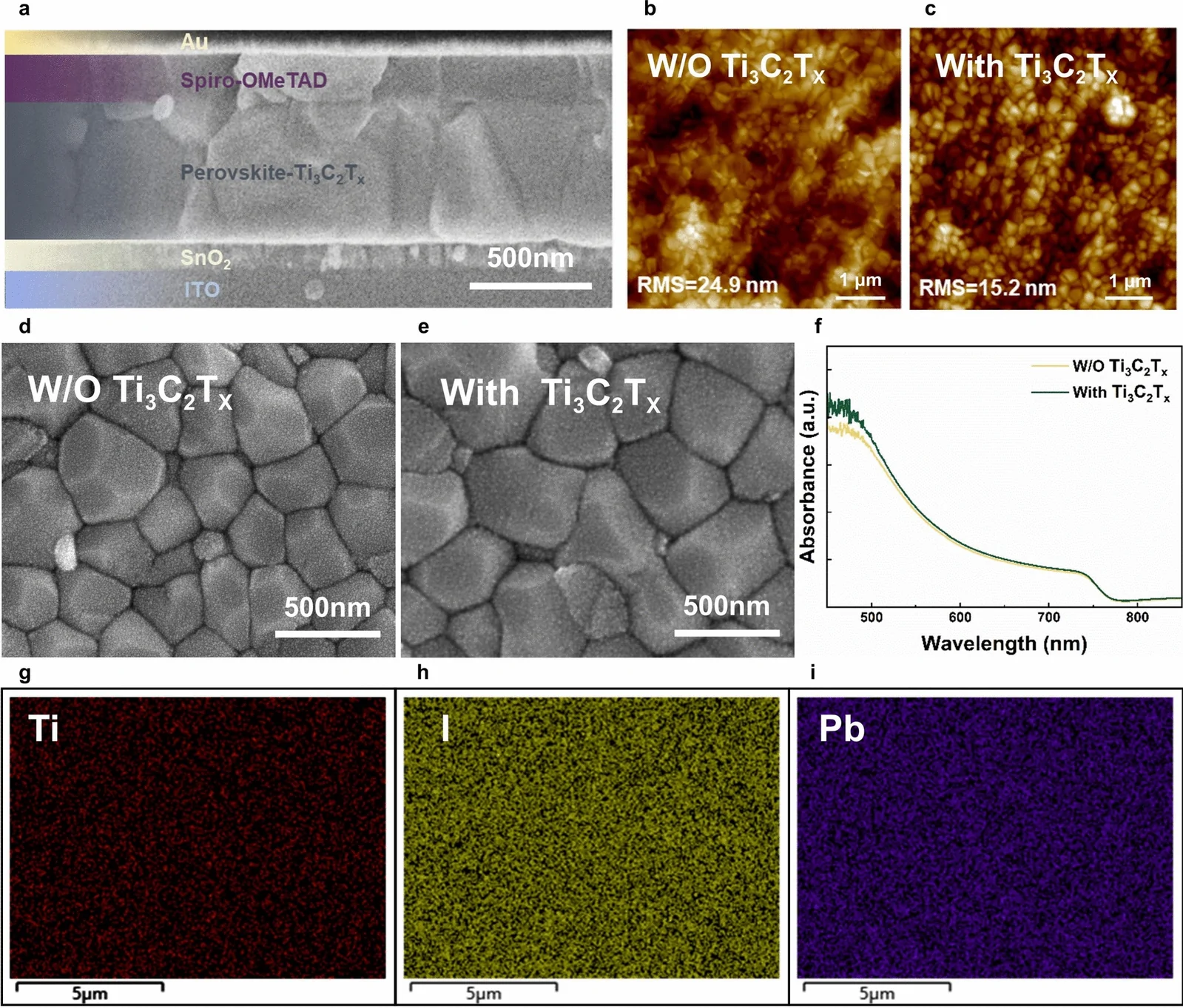
**a** Cross-sectional SEM image of Ti_3_C_2_T_X_-modified complete PSC. AFM images of perovskite films **b** without Ti_3_C_2_T_X_ and **c** with Ti_3_C_2_T_X_. SEM images of perovskite films **d** without and **e** with Ti_3_C_2_T_X_. **f** UV–Vis absorption spectra of perovskite films without and with Ti_3_C_2_T_X_. EDS spectra of **g** Ti, **h** I, **i** Pb in the Ti_3_C_2_T_X_-modified perovskite film

To investigate the multifunctional role of Ti_3_C_2_T_X_, we introduced Ti_3_C_2_T_X_ as an additive into perovskite precursor solution (Fig. S7). A series of perovskite films and complete PSCs containing different weight percent of Ti_3_C_2_T_X_ (0.01wt%, 0.03%, 0.05wt%) were prepared, with the PSC structure: ITO/SnO_2_/perovskite/Spiro-OMeTAD/Au. Figure 2a shows the cross-sectional SEM image of Ti_3_C_2_T_X_-modified PSC, where the perovskite layer was compact and uniform. This provides a solid foundation for excellent photovoltaic performance of PSCs.

Figure 2b, c presents AFM images of perovskite films' surface morphology. The root mean square roughness (RMS) of 0.03 wt% Ti_3_C_2_T_X_-modified perovskite film was 15.2 nm, compared to 24.9 nm for perovskite film without Ti_3_C_2_T_X_, indicating that the addition of Ti_3_C_2_T_X_ significantly improves the surface smoothness. Figure S8 further illustrates the effect of increasing Ti_3_C_2_T_X_ weight percent on the microstructure of perovskite film, showing that as the weight percent increases, RMS initially decreases and then increases, reaching a minimum at 0.03 wt%. An excess of Ti_3_C_2_T_X_ causes RMS to rise, likely due to the aggregation of surplus Ti_3_C_2_T_X_ at grain boundaries, which negatively affects the perovskite film's morphology. Next, we further explored the impact of Ti_3_C_2_T_X_ on the grain size of perovskite film using SEM (Fig. 2d, e). The results indicate that the grain size increases after the addition of Ti_3_C_2_T_X_. This improvement in morphology can be attributed to the excellent heat transfer ability of Ti_3_C_2_T_X_, which provides a stable thermal field for uniform grain growth, thereby enlarging the grain size.

To further verify the enhancement of perovskite film's light absorption capability by Ti_3_C_2_T_X_, ultraviolet–visible (UV–Vis) absorption spectroscopy was conducted. As shown in Fig. 2f, the perovskite films with and without Ti_3_C_2_T_X_ exhibited similar absorption onset points around 780 nm, consistent with typical wide-spectrum absorption characteristic of perovskite. But the Ti_3_C_2_T_X_-modified perovskite film displayed stronger absorption in the visible light region compared to control films, which can be attributed to grain growth and film morphology improvement. Larger grains can reduce grain boundary defects, increase crystalline order, and thus enhance light scattering and trapping effects.

Additionally, to confirm the distribution of Ti_3_C_2_T_X_, energy-dispersive spectroscopy (EDS) was employed to analyze key elements (Pb, I, Ti) in Ti_3_C_2_T_X_-modified perovskite film (Figs. 2 g-i and S9). The detection of Ti confirmed that Ti is uniformly distributed throughout the perovskite film and is not localized to any specific region. Due to the inability of Ti_3_C_2_T_X_ to enter the perovskite grain, it also means that Ti_3_C_2_T_X_ is distributed at the grain boundaries in the perovskite film. This distribution not only validates the successful incorporation of Ti_3_C_2_T_X_ but also suggests that it may act as an effective heat conduction pathway and defect passivator at grain boundaries, thereby improving the perovskite film's optoelectronic performance and stability.

**Fig. 3:**
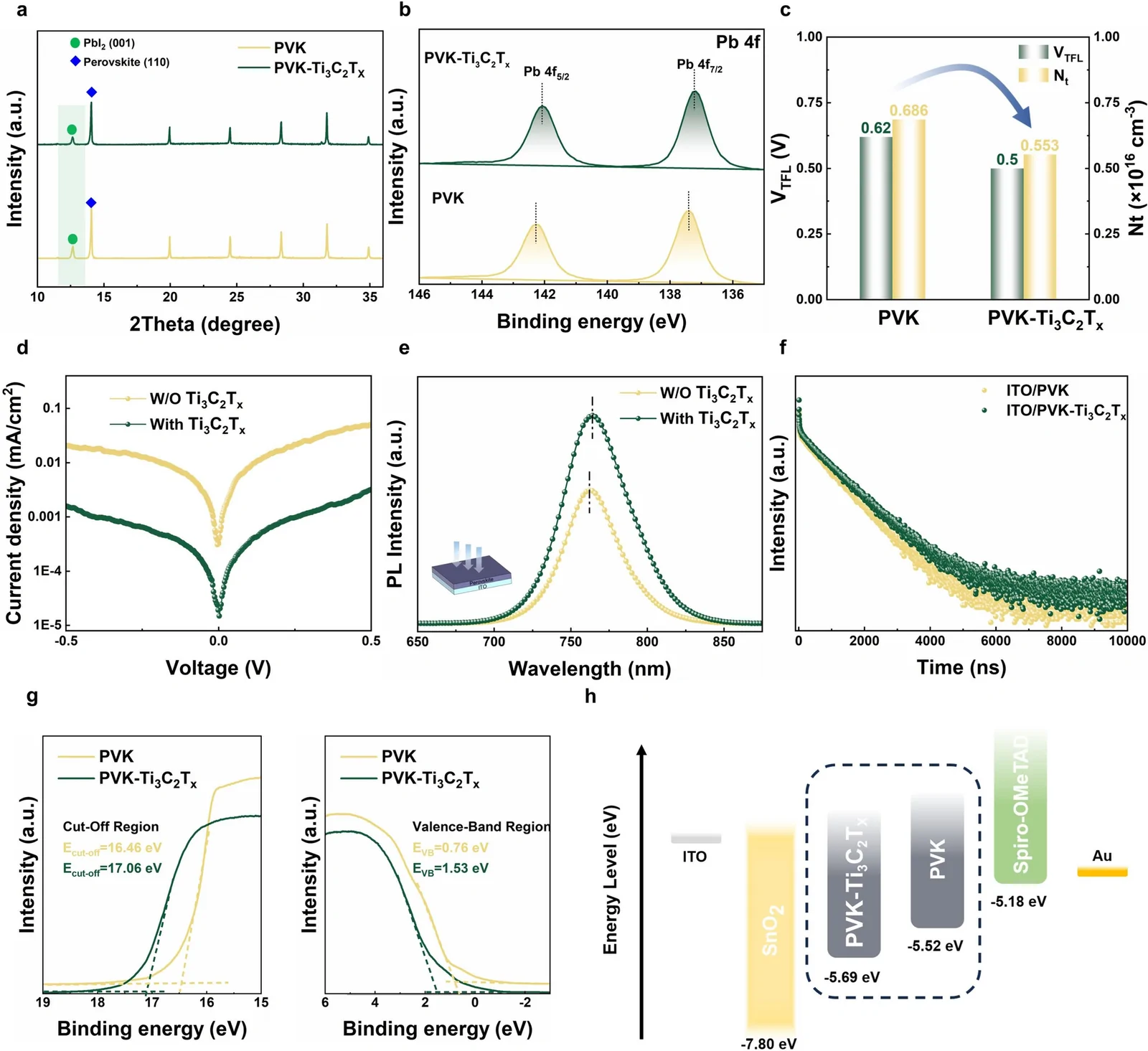
**a** XRD patterns and **b** XPS spectra of control and Ti_3_C_2_T_X_-modified perovskite films. **c** Recorded *V*_TFL_ and calculated N_t_ from SCLC of ITO/perovskite/Au and ITO/Ti_3_C_2_T_X_-modified perovskite/Au structures. **d** Logarithmic dark *J*-*V* characteristics of PSCs with and without Ti_3_C_2_T_X_. **e** Steady-state PL spectra and **f** TRPL spectra of ITO/perovskite and ITO/Ti_3_C_2_T_X_-modified perovskite structures. **g** UPS spectra of secondary electron cutoff and valence bands of without and with Ti_3_C_2_T_X_-modified perovskite films. **h** Scheme of energy level alignment for PSC

To further investigate the effect of Ti_3_C_2_T_X_ on the crystallinity of perovskite films, XRD analysis was conducted. As shown in Fig. 3a, the primary diffraction peaks of perovskite film appeared at 14.2°, 28.4°, and 31.82°, corresponding to (110), (220), and (310) crystal planes of perovskite, indicating good crystal orientation and crystallinity. With the introduction of Ti_3_C_2_T_X_, the intensity of PbI_2_ characteristic peak at 12.7° decreases, indicating that the addition of Ti_3_C_2_T_X_ helps reduce residual PbI_2_ in the perovskite film. This may be due to the interaction between the electron-rich terminal groups (O, OH, F) on the surface of Ti_3_C_2_T_X_ and the uncoordinated Pb^2+^ ions at grain boundaries or surface of perovskite, thus improving the crystallization process. To validate this hypothesis, XPS was used to analyze the perovskite films (Fig. 3b). The introduction of Ti_3_C_2_T_X_ led to a 0.2 eV shift of Pb 4*f* peak toward lower binding energy, indicating an interaction between Ti_3_C_2_T_X_ and Pb^2+^ ions in the perovskite. This interaction may result in a local redistribution of electrons at grain boundaries of perovskite, effectively passivating Pb^2+^ ions defects and further enhancing its optoelectronic performance. Moreover, XPS results further confirm that Ti_3_C_2_T_X_ are mainly distributed at grain boundaries, as only in this way can they interact with uncoordinated Pb^2+^ ions defects at grain boundaries.

Next, the space-charge-limited current (SCLC) measurement of pure perovskite devices with the ITO/perovskite/Au structure was performed, as shown in Fig. S10. By analyzing the different regions of* I*-*V* curve, we can better understand the trap density of perovskite films. The *I*-*V* curve initially showed a linear relationship at low bias, corresponding to the ohmic region. As the bias increases, the current raised non-linearly, entering the trap-filled limit (TFL) region. Finally, at high bias, the device reached the trap-free space-charge-limited current region. Based on the voltage at trap-filled limit (*V*_TFL_), the trap density in perovskite films can be calculated using the following equation [51]:

1 $$N_{t} = \frac{{2\varepsilon \varepsilon_{0} V_{{{\mathrm{TFL}}}} }}{{eL^{2} }}$$

*ε*₀ represents vacuum permittivity, *ε* is relative permittivity, and *V*_TFL_ is trap-filled limit voltage (obtained from the linear fitting intersection of *I*-*V* curve between the Ohmic region and the TFL region). *e* is electronic charge, and L is film thickness. As shown in Fig. 3c, after introducing Ti_3_C_2_T_X_, compared to control device, the *V*_TFL_ of modified device decreased from 0.62 to 0.5 V, and the trap density reduced from 0.686 × 10^16^ to 0.553 × 10^16^ cm^−3^. This result directly demonstrates that the introduction of Ti_3_C_2_T_X_ effectively passivates the defects and improves the quality of perovskite film. Moreover, the carrier mobility was also evaluated using SCLC measurements. A hole-only device with the structure ITO/PEDOT:PSS/perovskite/Spiro-OMeTAD/Au was fabricated, and the hole mobility was calculated using the SCLC Eq. S1. Compared with the control device (5.31 × 10^–5^ cm^2^ V^−1^ s^−1^), the hole mobility of the Ti_3_C_2_T_X_-modified device increased to 6.91 × 10^–5^ cm^2^ V^−1^ s^−1^ (Fig. S11). From the dark-state* J*-*V* curves of complete PSC (Fig. 3d), the dark current density significantly decreased after the perovskite film modified with Ti_3_C_2_T_X_, further confirming the reduction of defect states within the perovskite film.

Figure 3e presents the steady-state photoluminescence (PL) spectra of perovskite films with and without Ti_3_C_2_T_X_. By comparing the PL spectra, the Ti_3_C_2_T_X_-modified perovskite film showed a higher PL intensity, along with a slight redshift in the emission peak, suggesting that non-radiative recombination caused by defects has been significantly suppressed. To further explore the charge transport dynamics, time-resolved photoluminescence (TRPL) was conducted on the perovskite films with and without Ti_3_C_2_T_X_ (Fig. 3f). The data were fitted using a biexponential model:

2 $$y = A_{1} \exp \left( { - \frac{t}{{\tau_{1} }}} \right) + A_{2} \exp \left( { - \frac{t}{{\tau_{2} }}} \right) + y_{0}$$

3 $$\tau_{{{\mathrm{ave}}}} = \frac{{A_{1} \tau_{1}^{2} + A_{2} \tau_{2}^{2} }}{{A_{1} \tau_{1} + A_{2} \tau_{2} }}$$

where *τ*_1_ and *τ*_2_ represent short and long lifetimes, A_1_ and A_2_ represent decay amplitudes, while y_0_ is a constant related to baseline offset. The fitting parameters of TRPL are presented in Table S3. The average carrier lifetime *τ*_ave_ is calculated using *τ*_1_ and *τ*_2_. Compared with the control film (*τ*_ave_ = 848.50 ns), the *τ*_ave_ of Ti_3_C_2_T_X_-modified perovskite film increased to 911.91 ns.

To investigate the effect of Ti_3_C_2_T_X_ on the energy level alignment of perovskite films, we performed UPS measurements. From the UPS spectra (Fig. 3 g), it can be observed that the cutoff energy (*E*_cut-off_) of control film is 16.46 eV, whereas after modification with Ti_3_C_2_T_X_, the *E*_cut-off_ shifts 0.6 eV toward higher binding energy, indicating that the Ti_3_C_2_T_X_ modification results in a reduction of *W*_F_. According to relevant formulas:

4 $$W_{{\mathrm{F}}} = {21}.{22} - E_{{{\mathrm{cut}} - {\mathrm{off}}}}$$

*W*_F_ of control film is 4.76 eV, while that of Ti_3_C_2_T_X_-modified perovskite film is 4.16 eV. The *W*_F_ reduction of Ti_3_C_2_T_X_-modified perovskite film is primarily influenced by the surface functional groups of Ti_3_C_2_T_X_. The prepared Ti_3_C_2_T_X_ is rich in –OH groups, which possess strong electron-donating capability, thereby increasing the electron density of Ti_3_C_2_T_X_ [52]. Additionally, due to high electronegativity of –OH groups, charges transfer from Ti_3_C_2_T_X_ to oxygen terminal groups, resulting in electrostatic dipole effect and significantly reducing the *W*_F_ of perovskite film. Based on UPS results, the energy level arrangement is shown in Fig. 3 h. The HOMO energy level of Ti_3_C_2_T_X_-modified perovskite film is − 5.69 eV, whereas that of control film is − 5.52 eV. After modification, the HOMO energy level becomes deeper, approaching the HOMO energy level of SnO_2_ electron transport layer, thus facilitating photogenerated electrons extraction.

### Photovoltaic and Thermal Aging Performances of PSCs

**Fig. 4:**
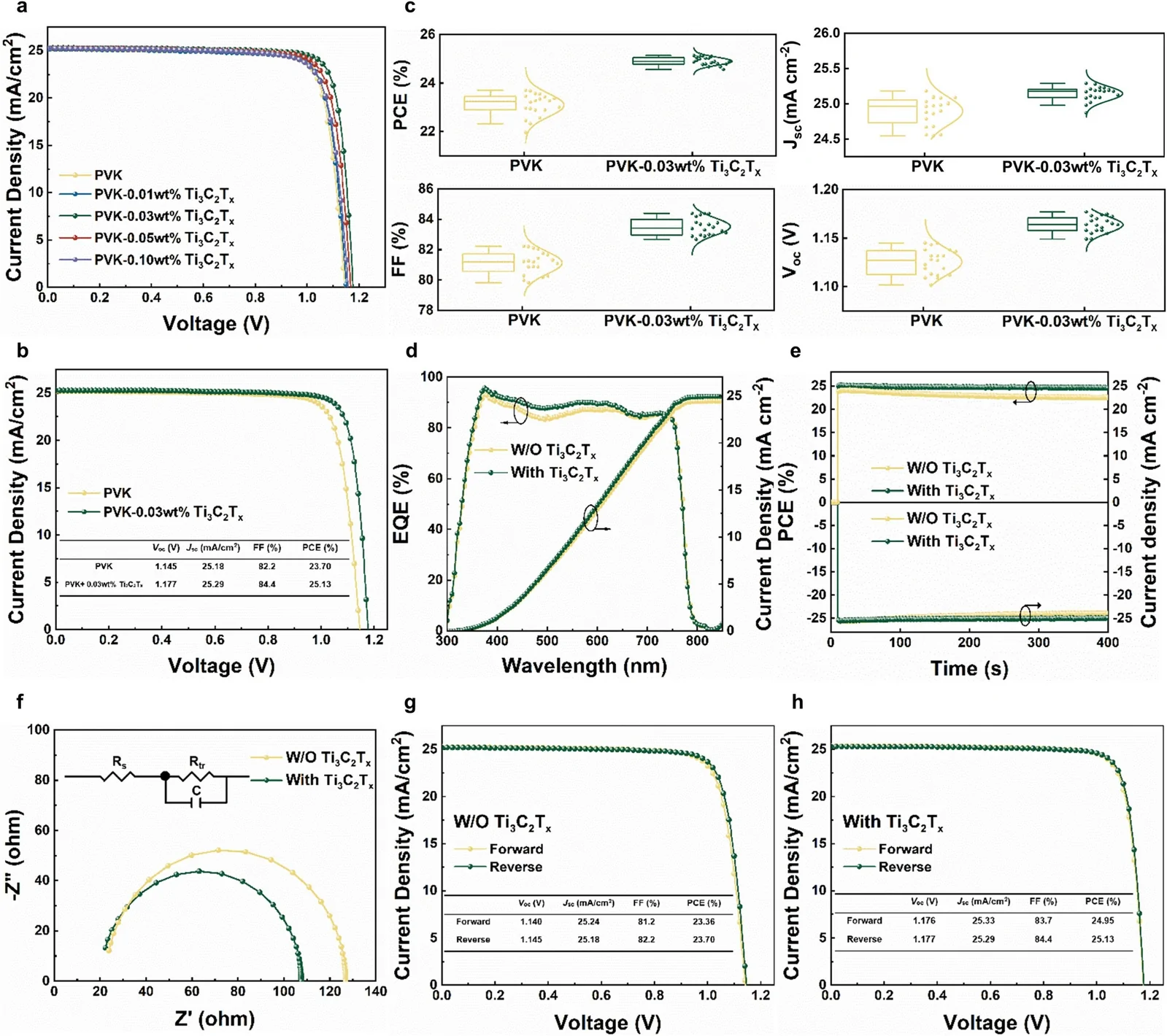
**a** *J*-*V* curves of PSCs with different weight percent of Ti_3_C_2_T_X_. **b** *J*-*V* curves of PSCs without and with Ti_3_C_2_T_X_. **c** PCE, *J*_sc_, FF, and *V*_oc_ statistics of 20 PSCs without and with Ti_3_C_2_T_X_. **d** EQE spectra and integrated current densities of PSCs without and with Ti_3_C_2_T_X_. **e** Steady-state PCE and *J*_sc_ outputs of PSCs without and with Ti_3_C_2_T_X_. **f** Nyquist plots of PSCs without and with Ti_3_C_2_T_X_ under one sun illumination. *J*-*V* curves of PSCs g without and **h** with Ti_3_C_2_T_X_ under forward and reverse scans

Figure 4a displays the *J*-*V* characteristic curves measured under one sun illumination for PSCs with different weight percent Ti_3_C_2_T_X_, and the relevant parameters are listed in Table S4. When the Ti_3_C_2_T_X_ weight percent reached 0.03 wt%, the PSC achieved the best PCE. Further increase in weight percent led to a decreasing trend in PCE. As shown in Fig. 4b, the 0.03 wt% Ti_3_C_2_T_X_-modified PSC exhibited a champion PCE of 25.13% with an open-circuit voltage (*V*_oc_) of 1.177 V, a short-circuit current density (*J*_sc_) of 25.29 mA cm^−2^, and a fill factor (FF) of 84.4%, whereas control PSC only achieved a PCE of 23.70% (*V*_oc_ = 1.145 V, *J*_sc_ = 25.18 mA cm^−2^, FF = 82.2%).

Figure 4c also displays histograms of PCE, *J*_sc_, FF, and *V*_oc_ distributions of 20 PSCs, which provided a visual comparison of photovoltaic parameters. Obviously, compared with control PSCs, 0.03 wt% Ti_3_C_2_T_X_-modified PSC exhibited the higher *V*_oc_, *J*_sc_, FF, PCE, and better reproducibility due to energy level matching, higher carrier extraction, and faster carrier transfer. The external quantum efficiency (EQE) spectra of PSCs with and without Ti_3_C_2_T_X_ were examined to confirm the accuracy of *J*_sc_ values of PSCs. As shown in Fig. 4d, the calculated integrated *J*_sc_ value of Ti_3_C_2_T_X_-modified PSC was 25.02 mA cm^−2^, which was in good agreement with the *J*_sc_ value obtained from experimental *J*-*V* measurement. Moreover, the Ti_3_C_2_T_X_-modified PSC exhibits higher EQE than the control PSC across almost the entire visible light spectrum. Additionally, to precisely analyze the output performance of PSC, the steady-state output at the fixed maximum power point (MPP) under one sun illumination for 400 s was conducted, as shown in Fig. 4e. The Ti_3_C_2_T_X_-modified PSC exhibited a stable efficiency of 24.60% and stable *J*_sc_ output of 24.69 mA cm^−2^, while the control PSC showed an output efficiency of 22.55% and stable *J*_sc_ output of 23.95 mA cm^−2^. Compared with control PSC, Ti_3_C_2_T_X_-modified PSC has more stable output capability.

Electrochemical impedance spectroscopy (EIS) is often used to analyze the carrier transport characteristics in PSCs. As shown in Fig. 4f, under one sun illumination, Ti_3_C_2_T_X_-modified PSC exhibited a lower transport resistance (*R*_tr_) compared to control PSC. Meanwhile, the recombination resistance (*R*_rec_) of Ti_3_C_2_T_X_-modified PSC was significantly increased in a dark environment (Fig. S12). These indicate that carrier recombination was effectively suppressed in Ti_3_C_2_T_X_-modified PSC and exhibited more effective charge transfer, thereby further improving the FF and PCE. This is mainly attributed to the significant reduction of defect states in perovskite film. Besides, the existence of hysteresis effects the PCE accuracy of PSCs. Under the reverse and forward scanning, the hysteresis of Ti_3_C_2_T_X_-modified PSC was smaller and can be ignored compared to control PSC, displayed in Fig. 4g, h. Therefore, these results demonstrate that Ti_3_C_2_T_X_ has more potential to improve the PSC performance and stability.

**Fig. 5:**
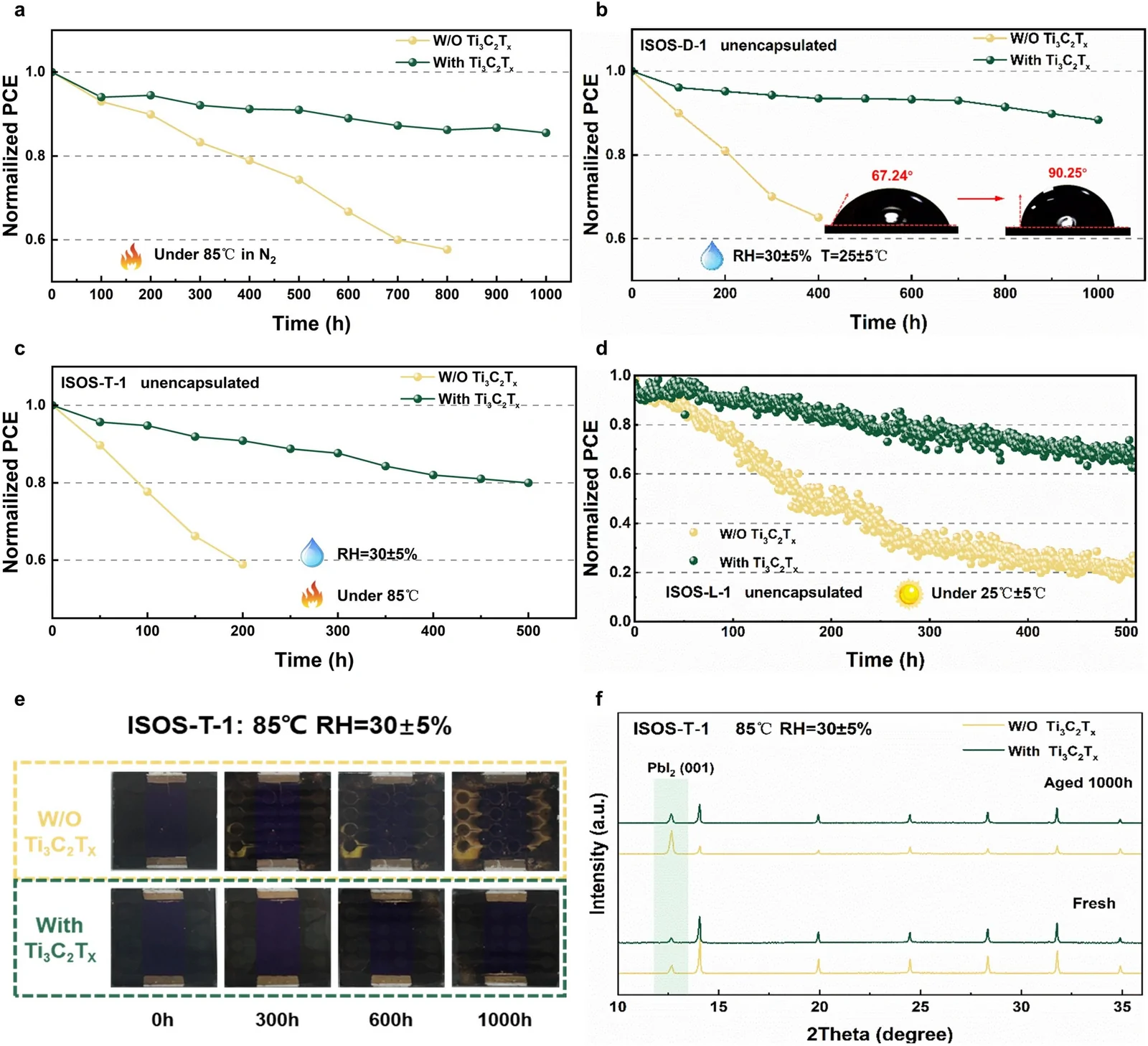
Normalized PCE variations of PSCs after aging at **a** 85 °C in N_2_, **b** room temperature and RH = 30 ± 5% with the inset illustrating the contact angle of perovskite film and **c** 85 °C and RH = 30 ± 5%. **d** Steady-state power output stability of PSCs operating at bias near maximum power output point under 25 ± 5 °C in N_2_. **e** Image of PSCs aging evolution under ISOS-T-1 conditions. **f** XRD patterns of perovskite films after aging under ISOS-T-1 conditions

To investigate the effect of Ti_3_C_2_T_X_ thermal management on the stability of unencapsulated PSCs, we conducted stability tests under three different conditions. 1) High-temperature aging test: After aging for 1000 h at 85 °C in N_2_, the Ti_3_C_2_T_X_-modified PSC maintained 86% of its initial PCE, while control PSC's PCE dropped to 57% after aging for 800 h (Fig. 5a). 2) Air aging test: In air at room temperature with 30 ± 5% relative humidity (ISOS-D-1), after 1000 h of aging, the Ti_3_C_2_T_X_-modified PSC retained 88% of its initial PCE, whereas the PCE of control PSC decreased to around 64% (Fig. 5b). 3) High-temperature and air aging test: Under conditions of 85 °C and 30 ± 5% relative humidity (ISOS-T-1), the Ti_3_C_2_T_X_-modified PSC maintained 80% of its initial PCE after 500 h of aging, while control PSC’s PCE dropped to 58% after 200 h of aging (Fig. 5c). Lastly, in MPP tracking tests conducted in N_2_ (ISOS-L-1), the Ti_3_C_2_T_X_-modified PSC retained 70% of its PCE after 500 h of operation, while control PSC's PCE dropped to just 20% (Fig. 5d).

These results clearly demonstrate that the introduction of Ti_3_C_2_T_X_ significantly enhances the overall stability of PSCs, making it one of the most effective strategies in the field of thermal management (Table S5). Moreover, this strategy substantially mitigates the poor thermal stability issue associated with using Spiro-OMeTAD as the HTL, achieving one of the longest lifetimes to date for fully solution-processed PVK/Spiro-OMeTAD devices under 85 °C aging conditions (Table S6). The improved thermal stability can be attributed to Ti_3_C_2_T_X_ creating an efficient heat conduction pathway within perovskite layer, effectively dissipating heat and reducing material degradation caused by heat accumulation. The improved humidity stability is likely related to hydrophobic surface groups of Ti_3_C_2_T_X_ derived during the HF etching process. Contact angle measurements showed that the water contact angle of Ti_3_C_2_T_X_-modified perovskite film increased from 67.24° to 90.25° (inset of Fig. 5b), enhancing the moisture resistance of perovskite film and reducing moisture-induced damage to PSCs. Further analysis of aged control PSCs under ISOS-T-1 condition revealed significant degradation in control PSC after 1000 h (Fig. 5e). XRD analysis showed a pronounced PbI_2_ peak in control PSC after aging, indicating severe perovskite decomposition, whereas the PbI_2_ peak in Ti_3_C_2_T_X_-modified PSC changed slightly, demonstrating excellent long-term stability (Fig. 5f).

## Conclusion

In summary, our research demonstrates that incorporating Ti_3_C_2_T_X_ nanosheets into perovskite layer significantly improves the thermal management and overall performance of PSCs. By constructing efficient heat dissipation pathways at grain boundaries, Ti_3_C_2_T_X_ effectively mitigates thermal accumulation. The stable operating temperature of PSCs can be reduced from 42.96 to 39.97 °C under one sun illumination. Beyond its exceptional thermal conductivity, Ti_3_C_2_T_X_ passivates defects in perovskite layer by interacting with uncoordinated Pb^2+^ ions, thereby reducing charge recombination. Additionally, Ti_3_C_2_T_X_ can also adjust the *W*_F_ to enhance the energy level alignment between the perovskite layer and other functional layers, thereby improving charge transfer and extraction. The Ti_3_C_2_T_X_-modified PSC achieved a significant PCE of 25.13% and demonstrated long-term stability under high-temperature and excellent operational stability. Even after 500 h of aging at 85 °C and RH = 30 ± 5%, the Ti_3_C_2_T_X_-modified PSC retained 80% of the initial PCE (control PSC retained 58% of initial PCE after 200 h of aging). Besides, the Ti_3_C_2_T_X_-modified PSC retained 70% of its PCE under 500 h MPP tracking in N_2_, while the PCE of control PSC dropped to just 20%. These results highlight the multifunction of Ti_3_C_2_T_X_ in thermal management and defect passivation, offering a promising approach to overcome the thermal instability of PSCs. However, despite the notable improvement in the thermal performance of PSCs upon Ti_3_C_2_T_X_ incorporation, challenges related to practical scalability remain. The cost and synthesis complexity of Ti_3_C_2_T_X_ may hinder its large-scale application. Future studies should focus on optimizing the synthesis routes to reduce cost and improve reproducibility. Moreover, alternative MXene materials with unique structures and electronic properties, such as Ti_2_CT_X_, for thermal management of PSCs may provide comparable or even superior performance.

## Supplementary Information

Below is the link to the electronic supplementary material.

Supplementary file1 (DOCX 2402 KB)
