# Supplementary material for: Multifunctional MXene for Thermal Management in Perovskite Solar Cells
Source: Nanomicro Lett. 2025 Aug 4;18:18. doi: 10.1007/s40820-025-01855-5 (PMC12321718; doi:10.1007/s40820-025-01855-5)
Supplement: Supplementary file 1 — Supplementary file1 (DOCX 2402 KB) [file 40820_2025_1855_MOESM1_ESM.docx]

Supporting Information for

**Multifunctional MXene for Thermal Management in Perovskite Solar Cells**

Zhongquan Wan^1,2^*, Runmin Wei^2^, Yuanxi Wang^1^, Huaibiao Zeng^2^, Haomiao Yin^1^, Muhammad Azam^1^, Junsheng Luo^1^*, Chunyang Jia^1^*

^1^National Key Laboratory of Electronic Films and Integrated Devices, School of Integrated Circuit Science and Engineering, University of Electronic Science and Technology of China, 611731 Chengdu, P. R. China

^2^Shenzhen Institute for Advanced Study, University of Electronic Science and Technology of China, 518110 Shenzhen, P. R. China

*Corresponding authors. E-mail: [zqwan@uestc.edu.cn](mailto:zqwan@uestc.edu.cn) (Zhongquan Wan); [luojs@uestc.edu.cn](mailto:luojs@uestc.edu.cn) (Junsheng Luo); [cyjia@uestc.edu.cn](mailto:cyjia@uestc.edu.cn) (Chunyang Jia)

# Supplementary Figures and Tables


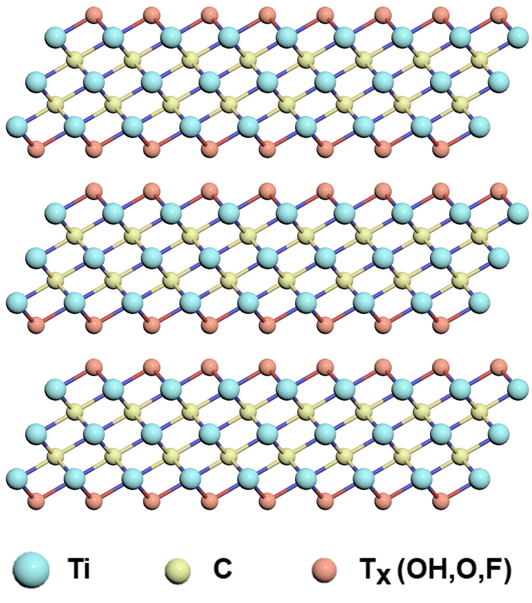


**Fig. S1** Schematic diagram of the multilayer Ti_3_C_2_T_X_


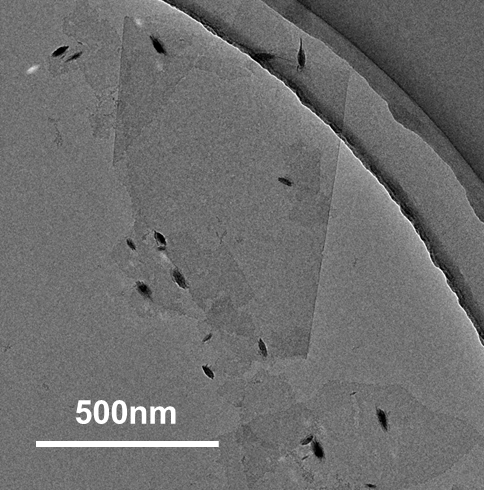


**Fig. S2** TEM image of single layer or few layer Ti_3_C_2_T_X_ nanosheets





**Fig. S3** XPS survey spectra of the Ti_3_C_2_T_X_

**Fig. S4** High-resolution XPS spectra of Ti 2p and C 1s for Ti_3_C_2_T_X_

**Fig. S5** Schematic diagram of films cooling test process: Transfer from 85 ℃ hot plate to room temperature cooling platform

**
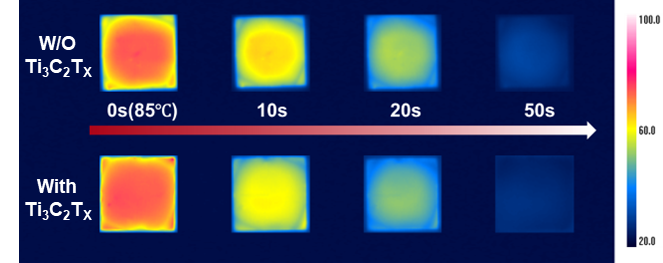
**

**Fig. S6** Infrared thermal images of ITO/perovskite and ITO/ Ti_3_C_2_T_X_-modified perovskite films under a cooling test


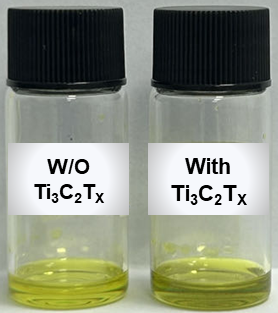


**Fig. S7** Comparison image of perovskite precursor solution without and with Ti_3_C_2_T_X_


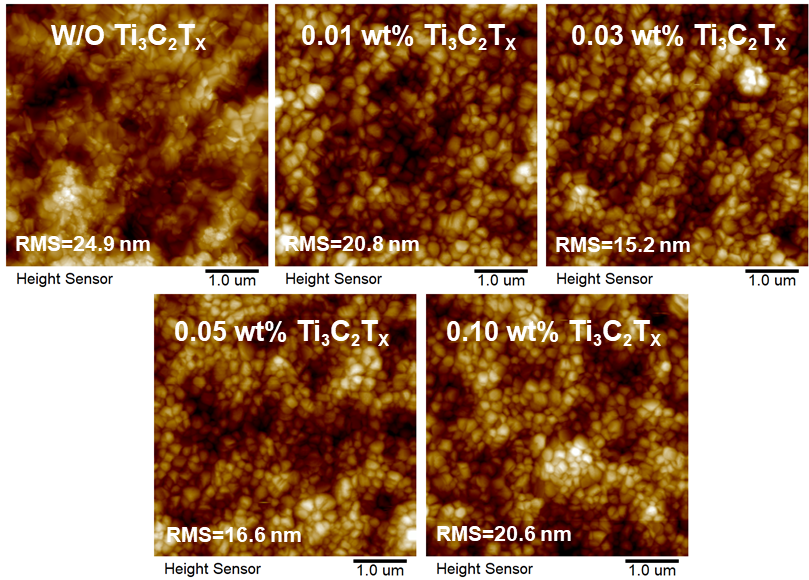


**Fig. S8** AFM images of perovskite films without and with 0.01 wt%, 0.03 wt%, 0.05 wt%, 0.10 wt% Ti_3_C_2_T_X_

**Fig. S9** EDS spectra of 0.03 wt% Ti_3_C_2_T_X_-modified perovskite film





**Fig. S10** *I*–*V* curves (log–log plots) of the pure perovskite device with the structure of ITO/PVK/Au and ITO/PVK-0.03 wt% Ti_3_C_2_T_X_/Au


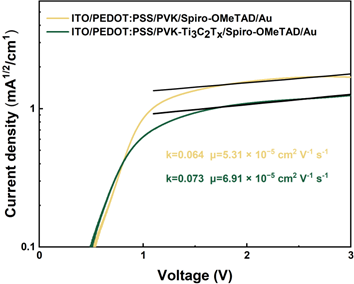


**Fig. S11** *J*–*V* curves (log–log plots) of the pure hole device with the structure of ITO/PEDOT:PSS/PVK-Ti_3_C_2_T_X_/Spiro-OMeTAD/Au





**Fig. S12** Nyquist plots of the PSCs with and without Ti_3_C_2_T_X_ under dark condition

**Equation S1** Carrier mobility calculation formula

$$J=\frac{9{\varepsilon_{0}\varepsilon}_{r}\mu{V_{\mathrm{eff}}}^{2}}{8L^{3}}$$

*J* is the measured current density, *V*_eff_ is the effective voltage defined as the difference between the applied voltage (*V*_app_) and the built-in voltage (*V*_bi_), L is the film thickness,$\text{ε}$_0_ is the vacuum permittivity, $\text{ε}$_r_ is the relative dielectric constant, and *μ* is the carrier mobility.

**Table S1** Comparison of thermal conductivities of various thermally conductive materials

| Thermally conductive materials | Thermal conductivity |
| --- | --- |
| h-BN | 51.1 W m^-1^ K^-1^ |
| Al_2_O_3_ | ~20-30 W m^-1^ K^-1^ |
| SiO_2_ | ~5 W m^-1^ K^-1^ |
| Zeolite | 1 W m^-1^ K^-1^ |
| **Ti_3_C_2_T_X_** | **55.8 W m⁻¹ K⁻¹** |

**Table S2** Input parameters for the simulation of PSC

| Input parameter | ITO | SnO_2_ | Perovskite | Spiro-OMeTAD | Au |
| --- | --- | --- | --- | --- | --- |
| Thickness (nm) | 100 | 50 | 500 | 350 | 50 |
| K (W·m^-1^·k^-1^) | 10 | 5 | 0.413 | 0.49 | 318 |
| h (W·m^-2^·k^-1^) | 3.1×10^8^ | - | - | - | 9.6×10^9^ |
| ρ (kg·m^-3^) | 7120 | 6950 | 4000 | 4128 | 19300 |
| C_p_ (J·kg^-1^·K^-1^) | 345 | 350 | 258 | 262 | 128 |

**Table S3** The fitting parameters of TRPL

| Simple | A_1_ | *τ*_1_ (ns) | A_2_ | *τ*_2_ (ns) | *τ*_ave_ (ns) |
| --- | --- | --- | --- | --- | --- |
| PVK | 0.943 | 7.628 | 0.344 | 868.744 | 848.50 |
| 0.03wt% Ti_3_C_2_T_X_-modified PVK | 0.824 | 9.276 | 0.369 | 931.975 | 911.91 |

**Table S4** The photovoltaic parameters of the PSCs

| PSC | *V*_oc_ (V) | *J*_sc_ (mA/cm^2^) | FF (%) | PCE (%) |
| --- | --- | --- | --- | --- |
| PVK | 1.145 | 25.18 | 82.2 | 23.70 |
| 0.01wt% Ti_3_C_2_T_X_-modified PVK | 1.151 | 25.22 | 81.6 | 23.69 |
| 0.03wt% Ti_3_C_2_T_X_-modified PVK | 1.177 | 25.29 | 84.4 | 25.13 |
| 0.05wt% Ti_3_C_2_T_X_-modified PVK | 1.167 | 25.28 | 82.4 | 24.30 |
| 0.10wt% Ti_3_C_2_T_X_-modified PVK | 1.157 | 25.24 | 80.9 | 23.62 |

**Table S5** Literature survey of the works on the thermal stability (85 ℃) of unencapsulated PSCs based on other thermal management strategies

| Year | Strategy | Champion PCE (%) | Aging time (h) | PCE/PCE^0^ (%) | Refs. |
| --- | --- | --- | --- | --- | --- |
| 2020 | Enhancing the thermal conductivity of Spiro with Al_2_O_3_ | 21.20 | 720 | 60 | [S1] |
| 2021 | Enhancing the thermal conductivity of Spiro with SiO_2_ | 22.29 | 1468 | 67 | [S2] |
| 2021 | Enhancing the thermal conductivity of PCBM with PS | 18.34 | 100 | 64 | [S3] |
| 2023 | Enhancing the thermal conductivity of Spiro with zeolite | 23.42 | 720 | 61 | [S4] |
| 2023 | Enhancing the thermal conductivity of PVK with h-BN | 19.8 | 300 | 85 | [S5] |
| **2025** | **Enhancing the thermal conductivity of PVK with Ti_3_C_2_T_X_** | **25.13** | **1000** | **86** | **This work** |

**Table S6** Literature survey of the works on the thermal stability (85 ℃) of unencapsulated PSCs based on PVK/Spiro-OMeTAD

| Device’s structure | Aging time (h) | PCE/PCE^0^ (%) | Refs. |
| --- | --- | --- | --- |
| ITO/SnO_2_/PCBM/PVK/Spiro-OMeTAD/Au | 1000 | 85.0 | [S6] |
| FTO/SnO_2_/PVK/Spiro-OMeTAD/Au | 27 | 85.0 | [S7] |
| FTO/SnO_2_/PVK/Spiro-OMeTAD/Au | 100 | 90 | [S8] |
| ITO/SnO_2_/PVK/Spiro-OMeTAD/Au | 450 | 92.0 | [S9] |
| FTO/bl-TiO_2_/mp-TO_2_/PVK/Spiro-OMeTAD/Au | 245 | 0 | [S10] |
| FTO/c-TiO_2_/mp-TO_2_/PVK/Spiro-OMeTAD/Au | 100 | 80.0 | [S11] |
| ITO/SnO_2_/MoO_3_/PVK/Spiro-OMeTAD/Au | 558 | 90.0 | [S12] |
| ITO/SnO_2_/TMPU/PVK/TMFS/Spiro-OMeTAD/Au | 500 | 94.2 | [S13] |
| ITO/SnO_2_/MgAc_2_/PVK/Spiro-OMeTAD/Au | 1250 | 80.0 | [S14] |
| FTO/TiO_2_/SnO_2_/PVK/Spiro-OMeTAD/Au | 200 | 60.0 | [S15] |
| FTO/SnO_2_/PVK/Spiro-OMeTAD/Au | 310 | 85.0 | [S16] |
| FTO/SnO_2_/PVK/Spiro-OMeTAD/Au | 500 | 92.4 | [S17] |
| FTO/SnO_2_/PVK/Spiro-OMeTAD/Au | 140 | 70.0 | [S18] |
| **ITO/SnO_2_/PVK-Ti_3_C_2_T_X_/Spiro-OMeTAD/Au** | **1000** | **86.0** | **This work** |

**Supplementary References**

1. K. Choi, J. Lee, H. Choi, G.-W. Kim, H.I. Kim et al., Heat dissipation effects on the stability of planar perovskite solar cells. Energy Environ. Sci. **13**(12), 5059–5067 (2020). <https://doi.org/10.1039/D0EE02859B>
2. F. Pei, N. Li, Y. Chen, X. Niu, Y. Zhang et al., Thermal management enables more efficient and stable perovskite solar cells. ACS Energy Lett. **6**(9), 3029–3036 (2021). <https://doi.org/10.1021/acsenergylett.1c00999>
3. Z. Zhang, Y. Tang, Y. Wang, Z. Zeng, R. Shi et al., Heat transfer enhancement of n-type organic semiconductors by an insulator blend approach. ACS Appl. Mater. Interfaces **14**(26), 30174–30181 (2022). <https://doi.org/10.1021/acsami.2c05503>
4. W. Wang, J. Zhang, K. Lin, J. Wang, B. Hu et al., Heat diffusion optimization in high performance perovskite solar cells integrated with zeolite. J. Energy Chem. **86**, 308–317 (2023). <https://doi.org/10.1016/j.jechem.2023.07.001>
5. Y. Yin, Y. Zhou, S. Fu, X. Zuo, Y.-C. Lin et al., Enhancing crystallization in hybrid perovskite solar cells using thermally conductive 2D boron nitride nanosheet additive. Small **19**(15), e2207092 (2023). <https://doi.org/10.1002/smll.202207092>
6. W. Song, L. Rakocevic, R. Thiruvallur Eachambadi, W. Qiu, J.P. Bastos et al., Improving the morphology stability of spiro-OMeTAD films for enhanced thermal stability of perovskite solar cells. ACS Appl. Mater. Interfaces **13**(37), 44294–44301 (2021). <https://doi.org/10.1021/acsami.1c11227>
7. W. Zhang, L. He, D. Tang, X. Li, Surfactant sodium dodecyl benzene sulfonate improves the efficiency and stability of air-processed perovskite solar cells with negligible hysteresis. Sol. RRL **4**(11), 2000376 (2020). <https://doi.org/10.1002/solr.202000376>
8. M. Chen, P. Li, C. Liang, H. Gu, W. Tong et al., Enhanced efficiency and stability of perovskite solar cells by 2D perovskite vapor-assisted interface optimization. J. Energy Chem. **45**, 103–109 (2020). <https://doi.org/10.1016/j.jechem.2019.10.006>
9. S. Tan, I. Yavuz, N. De Marco, T. Huang, S.J. Lee et al., Steric impediment of ion migration contributes to improved operational stability of perovskite solar cells. Adv. Mater. **32**(11), e1906995 (2020). <https://doi.org/10.1002/adma.201906995>
10. H. Zheng, G. Liu, C. Zhang, L. Zhu, A. Alsaedi et al., The influence of perovskite layer and hole transport material on the temperature stability about perovskite solar cells. Sol. Energy **159**, 914–919 (2018). <https://doi.org/10.1016/j.solener.2017.09.039>
11. K.-M. Lee, S.Y. Abate, J.H. Yang, W.-H. Chiu, S. Ahn et al., Facile synthesis of spiro-core-based hole-transporting material for high-performance and stable perovskite solar cells. Chem. Eng. J. **454**, 139926 (2023). <https://doi.org/10.1016/j.cej.2022.139926>
12. C. Liang, H. Gu, Y. Xia, Z. Wang, X. Liu et al., Two-dimensional Ruddlesden–Popper layered perovskite solar cells based on phase-pure thin films. Nat. Energy **6**(1), 38–45 (2021). <https://doi.org/10.1038/s41560-020-00721-5>
13. Z. Yi, X. Li, B. Xiao, Q. Jiang, Y. Luo et al., Dual-interface engineering induced by silane coupling agents with different functional groups constructing high-performance flexible perovskite solar cells. Chem. Eng. J. **469**, 143790 (2023). <https://doi.org/10.1016/j.cej.2023.143790>
14. M. Guli, Y. Zhang, R. Li, W. He, C. Lan et al., MgAc_2_-modified SnO_2_ electron transport layer for highly efficient and thermal stable perovskite solar cells. Nano Lett. **24**(45), 14183–14190 (2024). <https://doi.org/10.1021/acs.nanolett.4c02824>
15. Yukta, R.D. Chavan, A. Mahapatra, D. Prochowicz, P. Yadav et al., Improved efficiency and stability in 1, 5-diaminonaphthalene iodide-passivated 2D/3D perovskite solar cells. ACS Appl. Mater. Interfaces **15**(46), 53351–53361 (2023). <https://doi.org/10.1021/acsami.3c09887>
16. C. Shi, J. Li, S. Xiao, Z. Wang, W. Xiang et al., Alcohol-dispersed polymer complex as an effective and durable interface modifier for n-i-p perovskite solar cells. J. Energy Chem. **93**, 243–252 (2024). <https://doi.org/10.1016/j.jechem.2024.01.075>
17. Y.S. Shin, J. Lee, D.G. Lee, J.W. Song, J. Seo et al., Damp-heat stable and efficient perovskite solar cells and mini-modules with a *t*BP-free hole-transporting layer. Energy Environ. Sci. **18**(7), 3269–3277 (2025). <https://doi.org/10.1039/D4EE05699J>
18. K. Choi, J. Lee, H.I. Kim, C.W. Park, G.-W. Kim et al., Thermally stable, planar hybrid perovskite solar cells with high efficiency. Energy Environ. Sci. **11**(11), 3238–3247 (2018). <https://doi.org/10.1039/c8ee02242a>
